# To brace or not? The answer is “it depends”. Preliminary results from BrAIST

**DOI:** 10.1186/1748-7161-9-S1-O26

**Published:** 2014-12-04

**Authors:** Lori Dolan, Stuart Weinstein

**Affiliations:** 1 University of Iowa, Iowa City, IA, USA

## Background

Recent research suggests current indications for bracing in AIS result in significant over-treatment. Many patients are actually at low risk for significant progression. Others present at higher risk will benefit little from bracing.

## Aim

This study developed a simple, yet accurate, model of the risk of significant curve progression in AIS and the risk reduction associated with bracing.

## Methods

Data from 238 BrAIST subjects were used (91 observed, 147 braced). All met current indications for bracing (Cobb 20-40^ο^, Risser <3) and were followed until reaching a Cobb angle of >50<sup>ο</sup (failure) or until skeletal maturity. Logistic regression evaluated the relationship between initial variables, treatment and final outcome. The most predictive 3-variable model was chosen.

## Results

The overall failure rate was 31% after bracing and 52% after observation. Age, gender, Risser, Sanders' digital maturity stage (DMS), curve type and Cobb angle were all associated with outcome. DMS stages were more predictive of failure than Risser grade, even with age in the model. The best-fitting model included the DMS (1-2, 3, or 4+), Cobb angle, and treatment (p<0.0001, c statistic=0.841). Increasing Cobb angle was associated with increased risk of failure across all DMS’s; bracing significantly decreased the risk. In DMS 1-2, the risk of failure ranged from 73% (Cobb 20^ο^) to 93% (Cobb 39^ο^). Bracing reduced the risk to 50% and 84%, respectively. Risk was lower in DMS 3 patients, ranging from 36% (Cobb 20^ο^) to 76% (Cobb 40^ο^), reducing to 19% to 52% with bracing. The lowest risk of failure was noted at DMS 4+, ranging from 9% (Cobb 20^ο^) to 31% (Cobb 39^ο^), reducing to 3% to 15% with bracing.

## Conclusion

DMS stages in combination with Cobb angle at presentation accurately predict the natural history of AIS during the high-risk period. Bracing significantly altered the natural history. This model provides a simple, yet predictive model of the risk of curve progression and the decrease in risk due to bracing. These results can be used by clinicians and families to make evidence-based decisions concerning bracing for AIS, with the family choosing observation or bracing based on their own risk-benefit considerations.

